# Association among general health, personality traits, and headache severity in patients with migraine 

**DOI:** 10.22088/cjim.15.1.18

**Published:** 2024

**Authors:** Pardis Asadi, Angela Hamidia, Sara Mohammadnia, Ali Alizadeh Khatir, Ali Bijani, Soheil Ebrahimpour, Mir Saeid Ramezani

**Affiliations:** 1 Student Research Committee, Babol University of Medical Sciences, Babol, Iran; 2 Clinical research Development Unit of Shahid Yahyanezhad Hospital, Babol University of Medical Sciences, Babol, Iran; 3 Mobility Impairment Research Center, Health Research Institute, Babol University of Medical Sciences, Babol, Iran; 4 Social Determinants of Health Research Center, Health research Institute, Babol University of Medical Sciences, Babol, Iran; 5 Infectious Diseases and Tropical Medicine Research Center, Health Research Institute, Babol University of Medical Sciences, Babol, Iran; 6 Clinical Research Development Unit of Rouhani Hospital, Babol University of Medical Sciences, Babol, Iran

**Keywords:** Migraine, Personality type, Headache, General health

## Abstract

**Background:**

Because migraine is a common headache, finding ways to approach it better would be useful. So, studying the relation of dimensions of general health and personality types and pain severity in patients with migraine will be useful for fulfilling this aim.

**Methods:**

In this cross-sectional study, the number of patients with migraine headache studied in this study was 170. The checklists used in this study were migraine disability assessment (MIDAS), visual analog scale (VAS), Neuroticism-Extraversion-Openness (NEO FFI), and General Health Questionnaire (GHQ-28).

**Results:**

The average scores of general health dimensions in migraine sufferers with aura were higher than in migraine patients without aura. But this difference was significant only in the index of physical symptoms (P=0.02). There was a negative correlation between pain intensity and general health dimensions but it was not statistically significan. A positive correlation was observed between headache intensity and extroversion, which was significant (r=0.18 and P=0.01). The score of physical symptoms increases significantly with the increase of disability severity (P=0.007).

**Conclusion:**

According to the results, the severity of migraine disability, general health dimensions, and personality types in patients with and without Aura was not different. Also, general health dimensions and personality types were not associated with pain intensity and the severity of migraine disability.

Among the different neurological symptoms, headache is the one that is seen the most in patients (1) and the number of patients suffering from headaches globally is really high (2). Headaches are divided into two general groups. 1) Primary headaches and 2) Secondary headaches. Among these two, primary headaches are so much more frequent than secondary types; these two are classified into different types. Primary headaches are migraine, tension-type headache, cluster headache and other less common types such as continuous and new daily headaches. Secondary headaches are those headaches caused by another hidden disease (3).

Migraine as one of the primary headaches has recurrent episodes of headaches and can have some symptoms that can be associated with these headaches, such as photophobia, phonophobia, nausea and some other symptoms; these symptoms can last 4 to 72 hours. The concerning fact about migraine is that it can develop not only in young people but also in elderly people, but the occurrence decreases with aging (1, 4). There are two types of migraine; a migraine with aura, which is a focal neurological perception happening before the occurrence of headache that can have different symptoms such as visual, speech, sensory and motor symptoms, and a migraine without aura (5). 

Health is defined as "a state of complete physical, mental, and social well-being and not merely the absence of disease or infirmity", according to the World Health Organization (WHO) (6). In patients with migraine, improving the quality of health indexes such as physical condition, easing anxiety and stress, proper sleep hygiene and having enough good information about headaches can help them prevent and treat their pain (7, 8). It has been a long time since it was reported that there could be a relationship between personality types and migraine headaches (8). More severe migraines were more associated with personality disorders (9); in borderline personality disorder, patients experience more headaches and the pain is more severe than other migraine patients and that can make patients use drugs more than its safe level of usage (10, 11). Based on research findings and clinical observations, a series of psychological characteristics have been reported for people with migraine headaches, the most important of which are: anxiety, depression, perfectionism, ambition, order and extreme precision in daily activities and extreme sensitivity to life issues (12). In this regard, research findings show that people suffering from migraine headaches show a pattern of blaming and blaming themselves and others, and anger and aggression (12, 13). Another cognitive variable is coping strategies (12). The second factor related to the incidence of migraine headaches is personality traits (12, 13) The term migraine personality first emerged from various clinical observations; The most common personality types in people with migraine that have been reported so far are obsessive-compulsive personality disorder (23%), avoidant disorder (9.8%), borderline disorder (9.8%) (12). So, we decided to study if there was a correlation between personality types and health factors with migraine headaches.

## Methods

This cross-sectional study was conducted from December to March 2021 that 170 participants were chosen from people referring to health care centers related to Babol University of Medical Sciences. The sample size was calculated based on the following formula and considering the power values of 90%, r = 0.25, 150 people.

$\omega =\frac{1}{2}\mathrm{Ln}\frac{1+r}{1-r}$

$n=\frac{{\left(Z_{1-\frac{\alpha }{4}}+Z_{1-\beta }\right)}^{2}}{{\left(\omega \right)}^{2}}+3$

These participants were between 18 to 50 years old who had migraine and their disease was proven by a neurologist according to International Classification of Headache Disorders (ICHD). Those who did not want to take part in this study, illiterate people, those with major or emergency psychiatric diseases, and those with chronic diseases, cancers, dementia and mental retardation were excluded.

A check list was used for this study about their personal information that consists of age, sex, marital status, education level, job, health status. For studying migraine, migraine disability assessment (MIDAS) and other materials that were used in this study are visual analog scale (VAS) and for studying personality types, the Neuroticism-Extraversion-Openness Five-Factor Inventory (NEO FFI) and General Health Questionnaire-28 (GHQ-28) were used.

**Migraine disability assessment (MIDAS):** The MIDAS questionnaire was developed to evaluate the disability associated with headaches to improve the treatment of migraine. Patients with migraine responded to five questions to estimate how many days their activities have been restricted due to migraine in the past three months. After calculating their incapacity score by adding the total number of days for each of the five questions, the score was ranked as follows:

1. 0 to 5: MIDAS grade I, low or no disability
2. 6 to 10: MIDAS grade II, mild disability
3. 11 to 20: MIDAS grade III, moderate disability
4. 21 or higher: MIDAS grade IV, severe disability

The internal coherence, test-retest reliability, and validity of the questionnaire were evaluated in an independent populace overview of migraine patients. In expansion, the face validity, ease of use, and clinical utility of the questionnaire were assessed in a gather of 49 physicians who self-rated disease severity and required for consideration in the numerous types of migraine history. The test-retest Pearson correlation coefficient for the MIDAS add up to score was around 0.8. MIDAS scores approved against information on disability evaluations; the overall relationship between MIDAS and site-based estimation was 0.63. MIDAS scores moreover related with physicians' evaluations of treatment require (r = 0.69). Studies completed to date have appeared that the MIDAS questionnaire is consistent, reliable, valid, and relevant to clinician decision-making. This item supports compliance with treatment. The use of the MIDAS questionnaire progresses physician-patient communication almost headaches and may influence the well-being results of migraine patients (14).

**Visual analog scale (VAS):** The visual analog scale (VAS) could be a pain rating scale first utilized by Hayes and Patterson in 1921. Scores are based on self-reported measures of symptoms that are recorded with a single manually written check set at one point along the length of a 10-cm line that represents a continuum between the two closes of the scale—“no pain” on the cleared out conclusion (0 cm) of the scale and the “worst pain” on the proper conclusion of the scale (10 cm).10 Estimations from the beginning point (cleared out conclusion) of the scale to the patients' marks are recorded in centimeters and are deciphered as their pain. The values can be utilized to track pain progression for a patient or to compare pain between patients with comparative conditions. In spite of the fact that there's conflicting prove with respect to the advantage of VAS compared with other strategies for recording pain, it is still commonly utilized in clinical and domestic settings (15).

**General Health Questionnaire-28 (GHQ-28):** The GHQ-28 was created and presented as a screening device to distinguish those likely to have or to be at chance of creating psychiatric disarranges. The GHQ-28 may be a 28-item degree of the common mental well-being issues, counting substantial side effects (things 1–7), anxiety/insomnia (things 8–14), social dysfunctions (things 15–21), and serious misery (things 22–28). This survey has been interpreted into 38 dialects. The Persian form of the survey was created. They proposed a cutoff point of 23. Based on the Likert scoring strategy, the affectability and specificity of the survey were assessed at 70.5% and 92.3%, separately. The unwavering quality of the survey based on the esteem of Cronbach’s alpha was 87% (16).

**The NEO Five-Factor Inventory (NEO-FFI):** The NEO Five-Factor Inventory (NEO-FFI) could be an identity test that assesses an individual on five identity measurements, or the so-called Huge Five identity characteristics. These characteristics incorporate neuroticism, scruples, extraversion, pleasantness, and openness to modern encounters. The NEO-FFI was composed of 60 distinctive components (12 per characteristic). Costa and McCrae's 1978 distribution of an identity stock stamped the starting of the authentic improvement of the Changed NEO Identity Stock (NEO PI-R). The manual demonstrated the taking after inner textures for the NEO FFI: neuroticism= 0.79, extraversion= 0.79, openness= 0.80, suitability= 0.75, honesty= 0.83. The NEO-FFI was verified by Anisi et al. (17) employing a test of Iranian college understudies. The discoveries demonstrated that the scruples and neuroticism subscales had satisfactory unwavering quality values of 0.83 and 0.80, individually; whereas suitability and extraversion subscales had worthy unwavering quality values of 0.60 and 0.58, individually. Openness to modern encounters, be that as it may, did not show any inside relationship (0.39) (18).

**Data analysis:** After the study data was collected, it was entered into Excel software to prepare for statistical analysis. For the statistical analysis of this study, in the descriptive statistics section, central indices (mean, median, and mode) and dispersion indices (variance, standard deviation, range of changes, and coefficient of variation) for quantitative variables, as well as frequency, percentage and prevalence for qualitative data, were used. In the inferential statistics section, parametric (student's t-student, Chi-square) and non-parametric (Mann-Whitney, Kruskal-wallis) tests were used to check the study hypotheses after checking the normality of the data. All data analyses were done using SPSS Version 25 software. A significant level of p<0.05 was considered. This research was accepted by the Ethics Committee of Babol University of Medical Sciences with ethical code IR.MUBABOL.REC.1399.404.

## Results

In this study, 170 patients with migraine headaches who referred to Omid Polyclinic and Ayatollah Rouhani Hospital in Babol were evaluated in terms of disability caused by migraine, general health, personality type and pain scale. Among them, 101 (59.4%) clients were females. The average age of the patients was 32.83 ± 7.99 years with a median of 32 years, with a minimum age of 18 and a maximum of 50 years. The average duration of migraine in patients is 8.95 ± 7.73 years with a median of 7 years, with a minimum duration of 1 and a maximum of 33 years. The average pain intensity in the patients according to the VAS questionnaire was 6.29±2.20 with a median of 7; the minimum pain score was 1 and the maximum was 9. In examining the presence of aura in patients, 111 (65/3%) subjects did not mention the presence of aura before the onset of migraine headaches, and 59 people (34/7%) reported the presence of aura. 

Migraine disability assessment (MIDAS) showed that 6 (3.5%) individuals had little or no disability due to migraine. 10 (5.9%) subjects had mild disability, 28 (16.5%) had moderate disability, and 126 (74.1%) individuals had severe migraine disability. No significant correlation was observed between MIDAS and the presence of aura. Although the rate of severe migraine disability was higher in migraine patients both with aura (72.9%) and without aura (74.8%).

In the examination of general health and its different dimensions in patients with migraine in relation to the presence or absence of aura, the results showed that the average scores of general health and its dimensions in migraine sufferers with aura were higher than in migraine patients without aura. But this difference was significant only in the index of physical symptoms (P=0.02) (table 1). In examining the relationship between the personality types of migraine patients and aura, it was found that the average score of conscientiousness personality type in migraine patients with aura was 54.47±6.32 and in migraine patients without aura was 42.83±6.87 that the observed difference was statistically significant (P=0.02). However, in other personality types, no statistically significant difference was observed between migraine patients with and without aura (table 2). 

**Table 1:** The relationship between general health and its dimensions in migraine sufferers with the presence of aura

**Dimensions of general health**	**Without aura (n=111)**		**With aura (n=59)**		**P-value**
	**Mean**	**SD**	**Mean**	**SD**	
**Physical dysfunction**	8.60	4.09	10.47	4.92	0.02
**Anxiety**	7.45	4.86	8.76	4.91	0.07
**Social dysfunction**	7.62	3.44	7.58	3.88	0.92
**Depression**	5.23	4.45	5.09	5.02	0.21
**General health**	28.13	14.77	32.10	15.40	0.06

**Table 2:** The relationship between personality types in migraine sufferers with the presence of aura

**Personality types**	**Without aura (n=111)**		**With aura (n=59)**		**P- value**
	**Mean**	**SD**	**Mean**	**SD**	
**Neurosis**	36.85	7.60	33.63	6.26	0.50
**Extroversion**	30.95	5.33	61.64	4.74	0.40
**Openness**	36.89	4.29	36.42	4.04	0.49
**Adaptability**	41.14	5.40	40.25	5.41	0.30
**Conscientiousness**	42.83	6.87	54.47	6.32	0.02

There was a negative correlation between pain intensity and general health dimensions but it was not statistically significant; While the correlation between general health and its different dimensions was positive and significant (table 3). In the correlation study between pain intensity and personality types, a positive correlation was observed between headache intensity and extroversion, which was significant (r=0.18 and P=0.01). In other words, in extroverted personality types, the intensity of headache is greater. In other cases, there was no significant correlation between headache severity and personality types (table 4). In examining the severity of disability caused by migraine in different dimensions of general health, it was found that the score of physical symptoms increases significantly with the increase of disability severity (p=0.007). No statistically significant difference was observed between other dimensions of general health with severity of disability (table 5). In studying the correlation migraine disability level and personality types, a significant correlation wasn’t seen.

**Table 3:** The correlation of general health dimensions and headache severity in migraine patients

**General health dimensions**		**Physical appearance**	**Anxiety**	**Social dysfunction**	**Depression**	**General health**	**Headache severity**
**Physical symptoms**	**r**	1					
	**p**						
**Anxiety/insomnia**	**r**	0.68	1				
	**p**	<0.001					
**Social dysfunction**	**r**	0.40	0.53	1			
	**p**	<0.001	<0.001				
**Depression**	**r**	0.44	0.67	0.50	1		
	**p**	<0.001	<0.001	<0.001			
**General health**	**r**	0.80	0.90	0.69	0.77	1	
	**p**	<0.001	<0.001	<0.001	<0.001		
**Headache severity**	**r**	-0.08	-0.06	-0.09	-0.11	-0.12	1
	**p**	0.25	0.39	0.22	0.13	0.09	

**Table 4:** The correlation of personality types and headache severity in migraine patients

**Personality types**		**Physical appearance**	**Anxiety**	**Social dysfunction**	**Depression**	**General health**
**Headache severity**	**r**	-0.03	0.18	-0.09	-0.009	-0.005
	**p**	0.62	0.01*	0.21	0.90	0.94

*significant level of p<0.05

**Table 5:** The comparison between general health dimensions and Migraine Disability assessment score in migraine patients

**Migraine Disability** **Assessment score** **Dimensions of** **general health**	**NO Disability**		**Mild**		**Moderate**		**Severe**		**P-value**
	**Mean**	**SD**	**Mean**	**SD**	**Mean**	**SD**	**Mean**	**SD**	
**Physical symptoms**	4.67	1.21	8.30	4.99	8.07	3.63	9.81	4.55	0.007
**Anxiety**	5.83	4.83	6.90	4.33	7.64	4.28	8.14	5.09	0.53
**Social dysfunction**	7.83	1.72	7.30	2.21	8.00	3.88	7.66	3.70	0.94
**Depression**	5.55	5.00	6.25	4.50	4.92	4.14	5.18	4.75	0.83
**General health**	23.33	12.43	27.00	15.15	27.86	13.45	30.37	15.54	0.51

## Discussion

In this study, five different variables were studied in migraine patients; disability level because of migraine, general health, and different personality types in migraine patients with and without migraine and pain severity. Although migraine headaches are less common than tension headaches, they are more capable of causing disability in patients (19). In studying pain severity because of migraine, it was seen that 74.1% of patients had severe level of pain.

34.7% of participants reported aura before their migraine starts, but in a study done by Bener et al. it was reported that classic migraine which is a common type of migraine headaches is the reason of 15 to 20 percent of migraine headaches (20). This difference can have different reasons such as age distribution that in this study mean age was 32 and in Bener et al.’s study (20) it was 21 or the difference in the number of participants taking part in these two studies, but in a study done by Split et al., the prevalence of migraine with aura was 9% (21). 

In a study done by Wang et al., this prevalence was 6% and 1.2% of patients were experiencing both with and without aura migraines (22). But in a study done by Bahrami et al., the prevalence of classic migraine was reported 65.5% (23). These differences may be because of the difference in the society of participants in studies, because the prevalence of migraine with aura is higher in childhood and reaches its peak of presence in 17 (20). The disability caused by migraine in this study did not have significant difference between migraine patients with and without aura; but in patients with aura, the severe level of disability was seen in more patients than in those without aura (83 patients, 43 patients). The reason could be the aura that can make the patients refer to neurologists to ease the symptoms and because they will be treated sooner there would not be any significant correlation between the disability caused by migraine and the presence of aura. In this study, it was proven that the physical dysfunctions were more in migraine patients with aura, because aura can cause physical, neurological and behavioral symptoms (23). General health can help people adapt better to the environment and this is so important for patients with chronic disease like migraine patient who want to improve their daily functions (24).

Between migraine patients in this study the personality that was seen the most was conscientious. But in a study done by Goulart et al., there was a really close correlation between migraine and major depression, Obsessive–compulsive personality disorder, Generalized Anxiety Disorder (25). Maybe the high level of responsibility in obsessive people that causes internal conflicts can end in headaches. In a study by Garramone et al., it was reported that Neuroticism can affect levels of depression (26).In studying the correlation of general health and personality types with headache severity, it was seen that there was no significant correlation between headache severity and different health factors but the important thing was that, an increase in headache severity worsens general health and better general health status causes the headache severity decrease. The more severe the headache is, the sooner patients visit the neurologist so their general health is less affected. There is a significant positive correlation between headache severity and extroversion personality but this correlation with other personality types is negative. Yang et al. reported that risk of developing personality disorders is higher in those with migraine than other healthy normal people (9).

The difference between this study and Yang et al.’s study is in the measuring that was used in them in this study the took was NEO questionnaire and in Yang et al.’s study, it was DSM5.People with bipolar disorder and migraine experienced attacks every time their migraine would appear in a study that was done by Gordon-Smith et al. (27).There was not a significant difference in different types of personalities and the level of disability in migraine patients in this study.

In another study done by Toubaei et al., it was reported that it could be a correlation between personality types and migraine, in migraine patients some of personality type B indicators were more present (28). The findings proved that there were not any differences in disability severity, health factors and personality types in migraine patients with and without aura; and also, there were not any correlations between general health and personality types with headache severity and migraine disability.
